# Sculpting the Silent Intricacies: A Rare Triad of Syphilitic Aneurysms Unveiled

**DOI:** 10.7759/cureus.60944

**Published:** 2024-05-23

**Authors:** Ahmad W Haddad, Monique A Prince, Mohammad Kloub, Bereket Tewoldemedhin, Adeniyi A Adelakun, Deema Haddad, Omar Al-Radideh

**Affiliations:** 1 Internal Medicine, Saint Michael's Medical Center, Newark, USA; 2 Internal Medicine, Suburban Community Hospital, Lower Bucks Hospital, Bristol, USA; 3 Infectious Diseases, Saint Michael's Medical Center, Newark, USA; 4 Internal Medicine, Saint Mary's General Hospital, Saint Clare's Health, Passaic, USA; 5 College of Medicine, Jordan University of Science and Technology, Irbid, JOR; 6 Internal Medicine, University of Florida College of Medicine, Gainseville, USA

**Keywords:** triple aneurysms, intra-abdominal aneurysm, abdominal aneurysm, cardiovascular aneurysm, aortic aneurysm

## Abstract

This case report revolves around a 73-year-old male patient who initially sought medical attention due to left lower extremity weakness. Suspicions of a potential vascular etiology arose during the initial clinical examination, prompting further investigation. Unexpectedly, computed tomography (CT) imaging of the abdomen and pelvis revealed the presence of three giant aneurysms. Concurrently, positive syphilis titers were identified. The patient's presentation, marked by focal neurological deficits, unveiled the incidental discovery of a triad of aneurysms involving the distal abdominal aorta, right common iliac, and left common iliac. The neurological symptoms observed in the patient were attributed to the compression within the left common iliac artery, leading to compromised blood flow to the lower extremity. Alternatively, the neurological deficits could be linked to neurosyphilis or a combination of both factors. This case underscores the critical role of considering syphilis in patients presenting with neurological symptoms. The unique discovery of extensive aortic abnormalities through imaging studies, specifically CT angiography, emphasized the importance of such diagnostic tools in unraveling complex and potentially life-threatening vascular pathologies. Recognizing the diverse manifestations of syphilis in patients with vast neurological symptoms is crucial for timely diagnosis and multidisciplinary management. This case emphasizes the need to keep a high index of suspicion for syphilis in individuals who have widespread aortic anomalies together with neurological symptoms, to sum up. The triad of aneurysms discovered incidentally in this 73-year-old patient underscores the intricate interplay between vascular and neurological manifestations. The timely diagnosis and multidisciplinary management of both the neurological and vascular aspects of this unique presentation are essential for ensuring optimal patient outcomes.

## Introduction

Syphilis, a sexually transmitted infection caused by spirochete bacterium treponema pallidum, has been recognized as the "great imitator" due to its diverse clinical manifestations that mimic various other diseases. Despite the availability of effective antibiotics, the incidence of syphilis has seen a resurgence in recent years, often presenting with atypical and severe complications [1]. While the neurological complications of syphilis, known as neurosyphilis, are well-documented, the association with giant aortic aneurysms is a rare and formidable entity [2]. Most people with cardiovascular syphilis remain asymptomatic for 15-30 years following the original infection, with symptoms appearing in the fourth or fifth decade of life on average [3,4]. The objective of this report is to present the clinical, radiological, and therapeutic aspects of this distinctive case, shedding light on the interplay between tertiary syphilis and the development of multiple giant aneurysms and emphasizing the importance of considering syphilis in the differential diagnosis of complex vascular presentations. Aortitis is a rare complication of syphilis, especially in the United States.

## Case presentation

A 73-year-old male, previously healthy, arrived at the emergency department with a chief complaint of left lower extremity weakness. This weakness had begun a week prior and had progressively worsened since then. He described difficulty walking and standing for extended periods, coupled with numbness in his left foot. Interestingly, he denied experiencing dizziness, visual alterations, difficulty swallowing, or slurred speech. Additionally, he reported no chest pain, shortness of breath, or changes in bowel movements. Notably, he also mentioned no issues regarding his erections. He said that lately, he started feeling a strange pulsatile sensation along his left groin. The patient denied taking any medications, with no past medical history of diabetes or hypertension. The patient has been a smoker of three cigarettes daily for about 30 years with no alcohol use. He has never had any abdominal ultrasound (US). He was divorced and heterosexual. In the past 15 years after his divorce, he had three partners, used contraception consistently, and never had sexual intercourse with men. 

**Figure 1:**
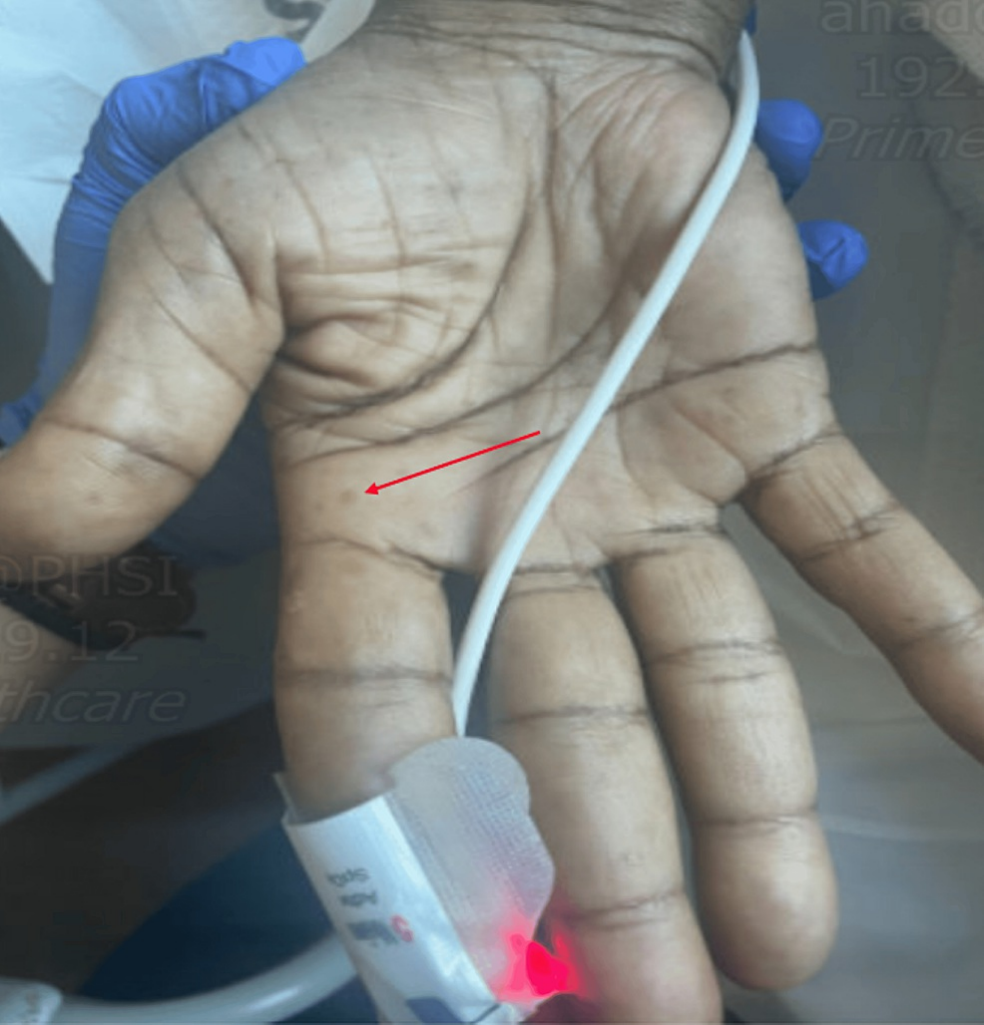
Hyperpigmentation nodules over the left hand with petechial nodules measuring <1 cm Red arrow shows a hyperpigmented nodule measuring less than <1 cm in diameter.

Vital signs were stable, with a normal blood pressure of 102/61 mmHg, a heart rate of 59 beats per minute, a respiratory rate of 14 breaths per minute, and normal O_2_ saturation. Upon physical examination, he was not in distress. A normal cardiac exam and a normal neurological exam showed normal motor strength and sensations over the bilateral lower extremities. Capillary refill was normal on both the left and right lower extremities. Abdominal examination showed a pulsatile mass with a positive bruit on auscultation. Skin examination revealed hyperpigmentation in both hands with petechial nodules measuring <1 cm, as shown in Figure 1. 

**Figure 2:**
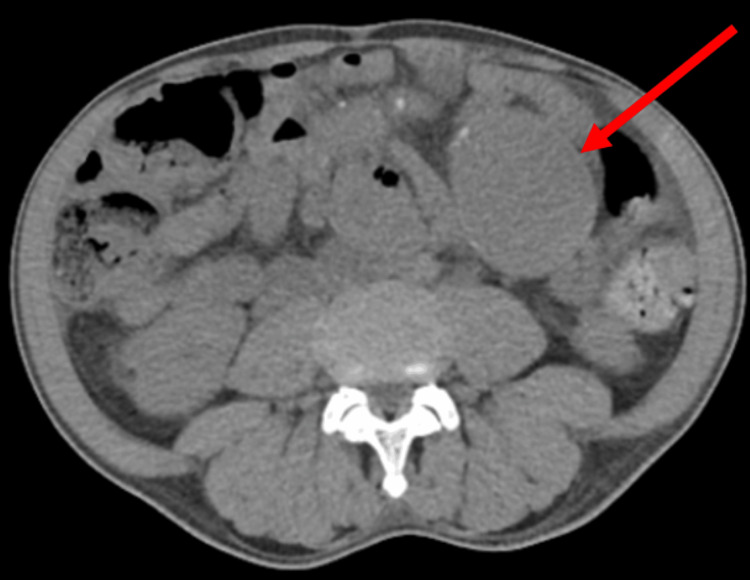
Red arrow highlights aneurysmal dilation of the distal abdomen measuring 5.5 cm

**Figure 3:**
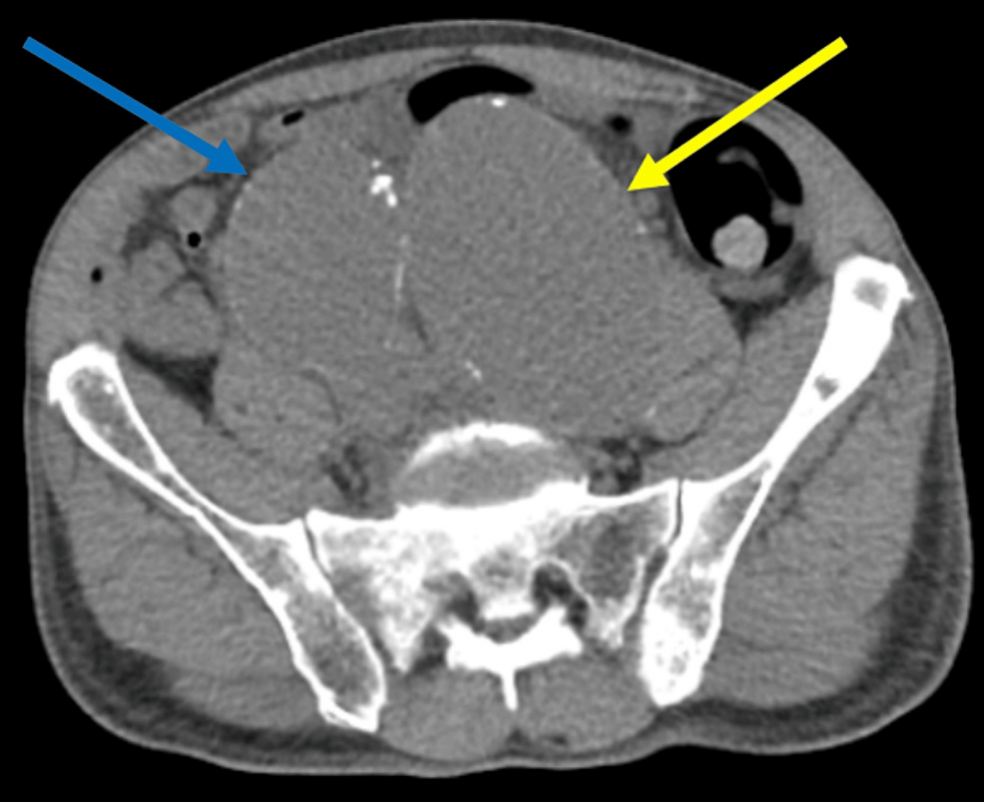
Yellow and blue arrows show left and right common iliac aneurysms measuring 7.0 and 5.3 cm, respectively

**Figure 4:**
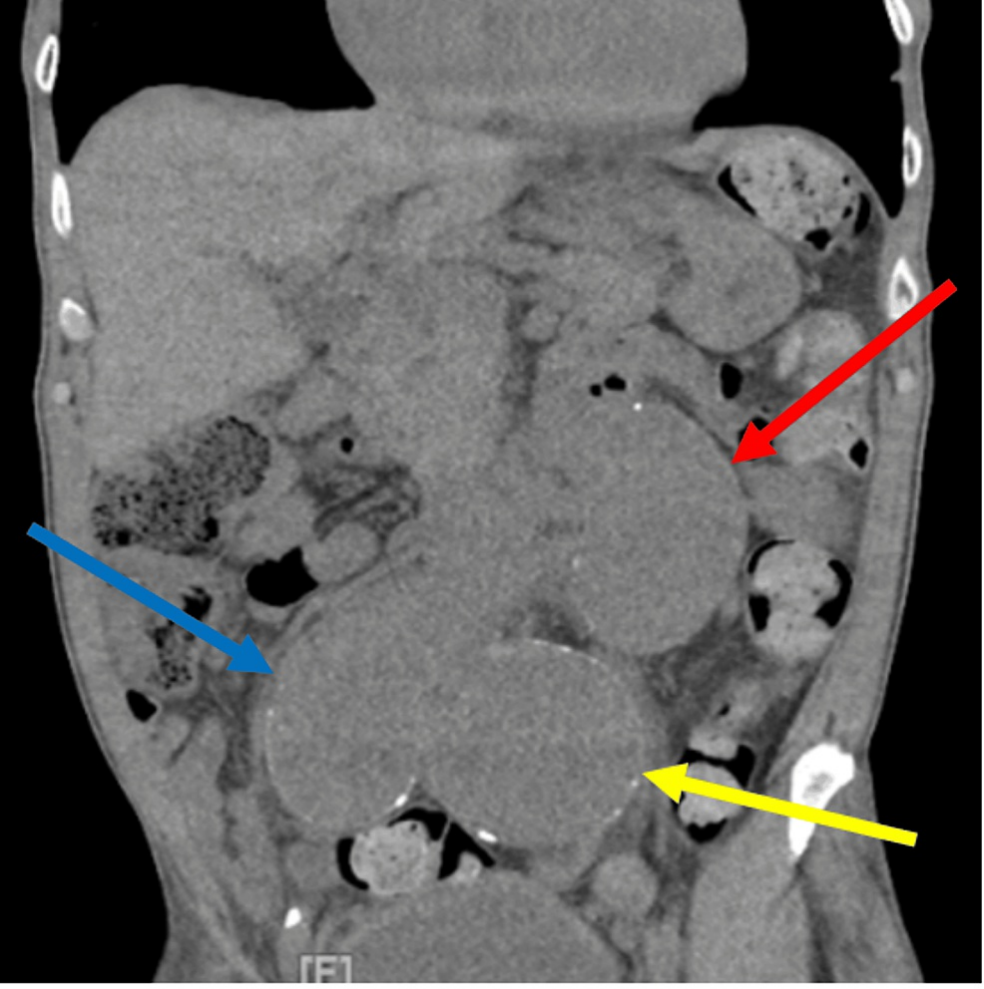
A view of the triple aneurysms; blue shows the right common iliac aneurysm measures 5.3 cm, the yellow arrow shows the 7 cm left iliac aneurysm, and the red arrow shows the distal abdominal aortic aneurysm, which measures 5.5 cm

In the emergency department, the complete blood count and complete metabolic panel were unremarkable, and his vitamin B12 and folate were normal. A computed tomographic scan showed aneurysmal dilation of the distal abdomen aorta measuring 5.5 cm, as shown in Figure 2. Other left and right common iliac aneurysms measuring 7.0 and 5.3 cm, respectively, are shown in Figure 3. A nice view of the triple aneurysms is shown in Figure 4. 

We ordered rapid plasma reagin (RPR), syphilis antibody determination (TP-PA), and fluorescent treponemal antibody absorption (FTA-ABS) tests, which came back 1:16, positive and reactive, respectively. A full STD panel was ordered, including HIV, viral hepatitis panel, gonorrhea, and chlamydia, which all subsequently came back negative. Treatment of IV penicillin G potassium 18 (million units) daily was started for this patient for 14 days. A follow-up for titers was done at 3,6,12 months and showed a decrease in titers by four folds, and the patient was scheduled for another RPR titer at 24 months. The patient was evaluated with cardiothoracic and vascular surgery, but he refused any intervention for his aneurysm. 

## Discussion

Tertiary syphilis, the non-contagious phase of syphilis, can arise many years after an untreated early or latent stage of *Treponema pallidum* infection. Approximately one-third of individuals with untreated latent syphilis developed tertiary syphilis in the pre-antibiotic period and occurred more frequently in men [5,6]. Currently, syphilitic aneurysms are deemed uncommon occurrences, with only a few case reports published in the past fifteen years. Nevertheless, syphilis still exists and persists at endemic levels in the United States, with 176,000 cases reported in 2021 [7]. Aortitis is the most common cardiovascular manifestation of tertiary syphilis, and approximately 10% of untreated patients develop critical complications [8]. These complications can lead to life-threatening events such as an aortic aneurysm. Aortitis has become infrequent, except in cases involving neurological complications in HIV-infected patients [6]. The pathogenesis involves spirochetes entering and resulting in obliterative endarteritis of the vasa vasorum, causing adventitial chronic inflammation and scarring. As a result, the aortic medium becomes ischemic, resulting in a varied loss of smooth muscle cells and related parenchyma. A fusiform or saccular aneurysm is caused by the loss of elastic rebound of the aorta due to destruction of the aortic medium, focal necrosis surrounded by palisading macrophages (microgrammes), and calcification that occurs inside fibrotic tissues [6]. 

Syphilitic aneurysms are often asymptomatic and are commonly discovered as incidental radiologic findings. Here, we demonstrate a surprising diagnosis of three syphilitic aneurysms in a patient presenting with neurologic dysfunction in the form of unilateral lower extremity weakness as the first manifestation. In 90% of cases, syphilitic aneurysms occur in the thoracic aorta, with less than 10% occurring in the abdominal aorta or its branches. Aneurysmal dilation may induce pressure on the nerve, and neurologic complications depend on the location of the aneurysm. It can occur at various levels of the nervous system, including peripheral nerve complications. In the last ten years, several syphilitic aortic aneurysm reports demonstrate varying clinical presentations, including simultaneous involvement of the brain, and spinal cord (neurosyphilis), or lungs (gummatous pulmonary syphilis), as well as rupture or dissection of the aneurysm [9].

In most cases, a single aneurysm is present, but in less than 10% of cases, there may be multiple [10]. Our review of the current literature yielded no findings related to triple syphilitic aneurysms. Large aneurysms may seldom present with signs of compression but carry a great risk for rupture with a high fatality rate. 

The diagnosis of tertiary syphilis can pose challenges due to potentially misleading clinical features. It can mimic other conditions, leading to diagnostic challenges and possible delayed treatment. Advancements in diagnostic modalities, such as serological tests like the rapid plasma reagin and the venereal disease research laboratory tests, are often used because of their high sensitivity [11]. However, they can be deemed as non-specific screening techniques since other conditions also generate positive results, including lymphoma, malaria, endocarditis, and viral infections [11]. Confirmatory tests include *Treponema pallidum* hemagglutination assay (TPHA) and fluorescent treponemal antibody absorption (FTA-ABS), with a specificity of 98% based on monoclonal antibodies and immunofluorescence [12]. CT and MRI have also improved the accuracy of diagnosis and early detection.

Our case stands out as unique, as in this case, the patient's initial complaint of left lower extremity weakness served as a gateway to uncovering a more intricate pathology involving the vascular system, and was found to have triple aneurysms in rare locations. Radiological imaging and syphilis antibody determination (TP-PA) tests confirm the presence of massive abdominal aortic and filial aneurysms in the setting of a negative HIV test. Periodic cardiac check-ups are necessary for high-risk individuals, such as sex workers, men having sex with men, intravenous drug users, and HIV-positive individuals, to prevent deadly results from early surgical intervention [13]. In the contemporary era, the clinical significance of syphilitic aortitis remains pertinent due to its potential to cause serious cardiovascular complications. Prompt recognition of subtle presentations is crucial for timely intervention to prevent mortality associated with its complications.

## Conclusions

Because of its unusual presentation, cardiovascular syphilis can be difficult to diagnose. A high index of suspicion in the right clinical context coupled with appropriate syphilis screening and the treponemal-specific test administered to high-risk individuals with thoracic aortic aneurysms can clinch the diagnosis. Early detection and treatment of tertiary syphilis with antibiotic therapy, followed by surgical or endovascular repair of syphilitic aortic aneurysms, can prevent fatal complications and mortality.

## References

[1] Jackman JD Jr, Radolf JD (1989). Cardiovascular syphilis. Am J Med.

[2] Willeford WG, Bachmann LH (2016). Syphilis ascendant: a brief history and modern trends. Trop Dis Travel Med Vaccines.

[3] Beppu K, Doi T, Hosokawa A (2014). Reprint of "Syphilitic aortic aneurysm missed on the chest radiography". Int J Cardiol.

[4] Paulo N, Cascarejo J, Vouga L (2012). Syphilitic aneurysm of the ascending aorta. Interact Cardiovasc Thorac Surg.

[5] Hofmann-Wellenhof R, Domej W, Schmid C, Rossmann-Moore D, Kullnig P, Annelli-Monti M (1993). Mediastinal mass caused by syphilitic aortitis. Thorax.

[6] de Araujo DB, Oliveira DS, Rovere RK, de Oliveira Filho UL (2017). Aortic aneurysm in a patient with syphilis-related spinal pain and paraplegia. Reumatologia.

[7] CDC CDC (2022). Sexually transmitted disease surveillance. Sexually transmitted disease surveillance.

[8] Pivatto Júnior F, Finkler BS, Torres FS, Schaefer PG, Sprinz E (2017). Aneurysm and dissection in a patient with syphilitic aortitis. Braz J Infect Dis.

[9] Chaudhary F, Faghihimehr A, Subedi Y, Hodanazari SM, Yousaf MN (2021). Syphilitic aortic aneurysm: a rare entity in the era of antibiotics. Cureus.

[10] Liu J, Yuan Q, Golamaully R, Gong T (2011). Syphilitic aortitis complicated by multiple aortic aneurysms: findings of multidetector CT. Int J Cardiovasc Imaging.

[11] Larsen SA, Steiner BM, Rudolph AH (1995). Laboratory diagnosis and interpretation of tests for syphilis. Clin Microbiol Rev.

[12] León LR, Mills JL (2009). Diagnosis and management of mycotic aneurysms. Curr Infect Dis Rep.

[13] Cocora M, Nechifor D, Lazar MA, Mornos A (2021). Impending aortic rupture in a patient with syphilitic aortitis. Vasc Health Risk Manag.

